# Adding a modified Lemaire procedure to ACLR in knees with severe rotational knee instability does not compromise isokinetic muscle recovery at the time of return-to-play

**DOI:** 10.1186/s40634-020-00302-1

**Published:** 2020-10-30

**Authors:** Leopold Joseph, Guillaume Demey, Thomas Chamu, Axel Schmidt, Alexandre Germain, Floris van Rooij, Mo Saffarini, David Dejour

**Affiliations:** 1 Lyon-Ortho-Clinic, Clinique de la Sauvegarde, Ramsay Santé, 29 Avenue des Sources, 69009 Lyon, France; 2 Sports Physiotherapy Centre, Lyon-Ortho-Clinic, Clinique de la Sauvegarde, 29 Avenue des Sources, 69009 Lyon, France; 3 Hôpital de la Croix-Rousse, Université Lyon 1, 103 Grande Rue de la Croix-Rousse, 69004 Lyon, France; 4 ReSurg SA, Rue Saint Jean 22, 1260 Nyon, Switzerland

**Keywords:** Anterior cruciate ligament reconstruction, Isokinetic tests, Lemaire procedure, Anterolateral complex, Pivot-shift

## Abstract

**Purpose:**

To determine whether isokinetic muscle recovery following ACLR using a hamstring tendon (HT) would be equivalent (non-inferior) in knees that had high-grade pivot-shift and adjuvant modified Lemaire procedure versus knees that had minimal pivot-shift and no adjuvant modified Lemaire procedure.

**Methods:**

We evaluated 96 consecutive patients that underwent primary ACLR. Nine were excluded because of contralateral knee injury, and of the remaining 87, ACLR was performed stand-alone in 52 (Reference group), and with a Lemaire procedure in 35 (Lemaire group) who had high-grade pivot-shift, age < 18, or genu recurvatum > 20°. At 6 months, isokinetic tests were performed at 240°/s and 90°/s to calculate strength deficits of hamstrings (H) and quadriceps (Q). At 8 months, patients were evaluated using IKDC, Lysholm, and Tegner scores.

**Results:**

Compared to the Reference group, the Lemaire group were younger (23.0 ± 2.5 vs 34.2 ± 10.5, *p* = 0.021) with a greater proportion of males (80% vs 56%, *p* < 0.001). The Lemaire group had no complications, but the Reference group had one graft failure and one cyclops syndrome. Strength deficits at 240°/s and at 90°/s were similar in both groups, but mixed H/Q ratios were lower for the Lemaire group (1.02 ± 0.19 vs 1.14 ± 0.24, *p* = 0.011). IKDC and Lysholm scores were similar in both groups, but Tegner scores were higher in the Lemaire group (median, 6.5 vs 6.0, *p* = 0.024).

**Conclusions:**

ACLR with a modified Lemaire procedure for knees with rotational instability grants equivalent isokinetic muscle recovery as stand-alone ACLR in knees with no rotational instability. For ACL-deficient knees with high-grade pivot-shift, a Lemaire procedure restores rotational stability without compromising isokinetic muscle recovery.

**Study design:**

Level III, comparative study.

## Introduction

Anterior cruciate ligament tears are often aggravated by concomitant injuries of the anterolateral complex that increase the rotational instability of the knee [5, 8, 22, 25]. If left untreated, anterolateral complex injuries could compromise outcomes of anterior cruciate ligament reconstruction (ACLR), particularly risks of re-tear or subsequent meniscal injury in patients returning to pivoting sports [19].

Several techniques have been described for lateral extra-articular tenodesis (LET) of the anterolateral complex, for which the indications continue to evolve [16], the present consensus being patients aged under 30 and/or that have concomitant medial meniscus tears [6, 35]. The Lemaire procedure, popularised in the 1980s [26], with variants by Christel and Djian [7], as well as Ntagiopoulos and Dejour [35], proved effective in ACLR in terms of biomechanics [33], safety [36], and reproducibility [35]. This procedure has shown benefits in patients with high risks of re-tear or those participating in pivoting sports [16, 27], by limiting translation within the lateral compartment [15, 27, 39, 47], reducing pivot-shift [16, 30, 32, 47], and protecting the graft and meniscus [17, 18, 30], with little or no side effects [36].

Isokinetic evaluation of muscle strength recovery is becoming one of the primary tools to evaluate the safety of return-to-play (RTP) after ACLR [10, 11]. To the authors’ knowledge there are no published studies that evaluated muscle recovery at the time of RTP following ACLR with Lemaire extra-articular tenodesis. The purpose of this study was therefore to determine whether isokinetic muscle recovery following ACLR using a hamstring tendon (HT) would be equivalent (non-inferior) in knees that had high-grade pivot-shift and adjuvant modified Lemaire procedure versus knees that had minimal pivot-shift and no adjuvant modified Lemaire procedure. The hypothesis was that an extra-articular modified Lemaire procedure restores rotational stability of the knee without increasing isokinetic muscle deficit at the time of RTP.

## Methods

### Study design

**Fig. 1:**
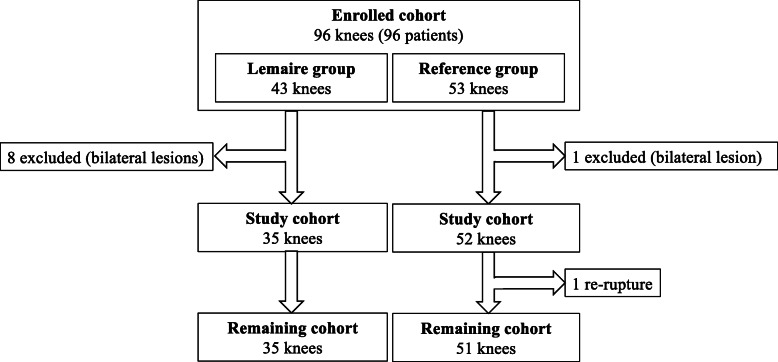
Flowchart

The authors retrospectively collected the records of 96 consecutive patients that underwent primary ACLR using HT autografts, with a Lemaire procedure (Lemaire group) or without a Lemaire procedure (Reference group), between March 2016 and May 2018 at a single center. All patients had subjective instability and functional impairment, confirmed by a positive Lachman test and/or pivot-shift test (PST). The decision to perform to a modified Lemaire procedure was discussed with the patients in advance and was based primarily on the following indications: clunk or gross PST, patient aged under 18 years, or > 20° genu recurvatum. Patients that had severe concomitant ligament injury (*n* = 0) or history of intra- or extra- articular surgery on the ipsi- or contralateral knee (*n* = 9) were excluded from this study (Fig. 1). This left a cohort of 87 patients, all of whom provided written informed consent for the use of their data and images for research and publishing purposes. This study was also approved by the institutional review board in advance (COS-RGDS-2020-03-006-DEJOUR-D).

### Clinical assessment

The following clinical assessment was performed pre- and post-operatively: The International Knee Documentation Committee (IKDC) functional score [23], Lysholm knee scale [28], and Tegner activity score [41] were recorded by an independent clinician. Anterior Cruciate Ligament-Return to Sport after Injury (ACL-RSI) scale [3] was also assessed post-operatively. Anteroposterior knee laxity was assessed using ‘static’ and ‘dynamic’ measurements of anterior tibial translation (ATT) on ‘true lateral view’ radiographs, superimposing the posterior femoral condyles. The ATT was defined as the distance between two lines parallel to the posterior tibial cortex: the first tangent to the posterior aspect of the medial tibial plateau, and the second tangent to the posterior femoral condyles [12, 35], Static ATT was measured on monopodal weight bearing radiographs with the knee flexed at 20°. Dynamic ATT was measured using the Telos™ stress radiography device (Metax, Hungen, Germany) with a constant anterior force of 150 N, and the side-to-side difference between the injured knee and healthy knee was calculated. A side-to-side difference of 9 mm was considered a complete ACL rupture [13]. Rotational laxity was determined with the PST by the senior surgeon (DHD), by applying a valgus force to the tibia in internal rotation while flexing the knee from full extension. The results were assessed using the criteria of the IKDC as none (0), glide (+ 1), clunk (+ 2) or gross (+ 3). The PST was considered high-grade if it indicated clunk or gross. Meniscal and ligament tears were assessed on magnetic resonance images (MRI).

### Surgical technique

All operations were performed under general anesthesia with a tourniquet placed high on the thigh. The graft was harvested from the hamstrings to form a 4-strand configuration using the gracilis and semitendinosus, which were left attached on their tibial insertions then doubled and sutured together to achieve appropriate length and thickness for the size of the patient. Meniscal tears and ligament status were further confirmed arthroscopically, by direct vision and palpation with a probe, at the time of ACL reconstruction. Meniscal treatments were performed when necessary, either by suture repair (Fast-Fix 360, Smith&Nephew, Memphis, TN) in stable lesions in zone 0 or 1 or by partial meniscectomy in unstable lesions or in those in zones 2 or 3 [9]. The femoral tunnel preparation was performed using an outside-in guide for femoral tunnel placement. For the tibial tunnel, a standard 55° angulation guide was used. The graft was passed from the tibia to the femur and fixed with Ligafix interference screws (SBM, Lourdes, France), with a composition of tri-calcium phosphate (TCP) of 30% for femoral tunnel screws and of 60% for tibial tunnel screws.

**Fig. 2:**
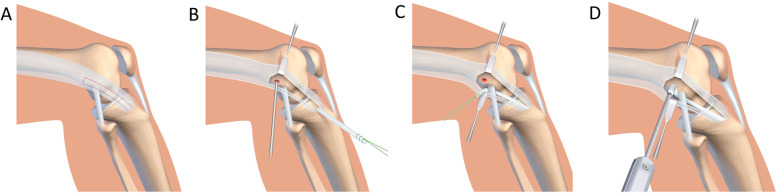
**a** The iliotibial band (ITB) was identified and incised to create a 10 mm × 80 mm strip originating from Gerdy’s tubercle (GT). **b** It was proximally sectioned, and whip stitched. **c** The ITB graft was then passed under the lateral collateral ligament (LCL), in a tunnel separate to that of the ACLR graft, at the isometric point. **d** The ITB graft was then fixed with an absorbable interference screw of a constant diameter of 7 mm

The modified Lemaire procedure was performed in 35 knees using the following method [35]. The knee was prepared and draped in the 90° flexed position (Fig. 2). The iliotibial band (ITB) was identified and incised to create a 10 mm × 80 mm strip originating from Gerdy’s tubercle (GT). It was proximally sectioned, and whip stitched. The ITB graft was then passed under the lateral collateral ligament (LCL), in a tunnel separate to that of the ACLR graft, at the isometric point. The ITB graft was then fixed with an absorbable interference screw of a constant diameter of 7 mm (SBM, Lourdes, France).

### Postoperative rehabilitation

The rehabilitation protocol was divided into three phases:

- Phase 1 (0–6 weeks): The ROM goals were to obtain full extension and 90° of flexion at 3 weeks, then 120° of flexion at 6 weeks. Partial weight-bearing (50% body weight) was allowed during the first 3 postoperative weeks if the preoperative static ATT was < 5 mm, [14] and progressive full weight-bearing was allowed between 3 and 6 weeks.
- Phase 2 (6–12 weeks): The aims were to get full ROM, increase muscular strength and knee stability. At the end of this phase, the patient was expected to walk quickly and climb stairs.
- Phase 3 (3–8 months): The aim was to regain the normal muscle strength and gradual return to sport. This phase consisted of heavy resistance strength training and exercises depending on type of sport practiced.

### Isokinetic tests

Isokinetic tests were performed by 2 physiotherapists (TC, AG), blinded to the study design, at 6–8 months follow-up, as part of a rehabilitation program to enable patients to achieve specific goals for return to sport at 9–12 months. The tests were performed using a CON-TREX system (Physiomed, Schnaittach, Germany) at the same rehabilitation center following a standardized protocol. The CON-TREX isokinetic dynamometer has demonstrated moderate-to-high reliability for both the knee extensor and the flexor muscle groups [29]. The patients warmed up using an ergometric bicycle for 15 min prior to the isokinetic tests. The subject was then seated on the dynamometer, stabilized by thoracic and lumbar straps, with a short tibial support on the tested leg, and a fixed contralateral leg. Testing started with the contralateral side to determine speed and amplitude.

Adequate preparation was done at submaximal intensity to familiarize patients, in concentric mode, to gain appreciation of the speeds and verify the patients’ understanding of the exercise. The first test was performed assessing the hamstrings and quadriceps muscle groups both in concentric and eccentric mode at an amplitude of 20°-90° of flexion completing 3 repetitions at 90°/s and 15 repetitions at 240°/s [20]. Between every test there was a rest period of 90 s. The second test was performed assessing the hamstrings at an amplitude of 90°-20° of flexion completing 3 repetitions at 30°/s in eccentric mode to evaluate recovery of the hamstring tendons. The percentage deficits of the healthy side compared to the contralateral side were collected for each muscle group (hamstrings, H; quadriceps, Q) and each angular velocity. The H/Q ratios were calculated for concentric strength at 240°/s and at 90°/s, as well as mixed speeds (H30/Q240), since they closely represent the biomechanics of athletes during sports [10].

### Statistical analysis

An a priori sample size calculation for the principal goal of the study indicated that 35 patients per group were needed to detect a minimal clinically important difference in isokinetic ratios of 5.75% [2] with a standard deviation (SD) of 11% and a statistical power of 0.80. The Shapiro–Wilk test was used to verify normality of distributions. Continuous variables were compared using unpaired t-tests or Mann-Whitney tests. Categorical variables were compared using Chi-square tests or Fisher’s exact tests. Statistical analyses were performed using R version 3.5.2 (R Foundation for Statistical Computing, Vienna, Austria). *P* values < 0.05 were considered statistically significant.

## Results

Of the initial cohort of 87 patients, 1 underwent revision surgery due to graft failure, leaving a final cohort of 86 patients, of which 35 were in the Lemaire group and 51 were in the Reference group (Table 1). Patients in the Lemaire group were younger (23.0 ± 2.5 years vs 34.2 ± 10.5 years, *p* = 0.021) with a greater proportion of males (80% vs 56%, *p* < 0.001), and had a significantly better flexion range (*p* = 0.022), even though 1 patient only had 60° of flexion. The two groups had a similar prevalence of meniscal lesions (*p* = 0.368) and types of meniscal treatments (*p* = 0.711 and *p* = 0.599) (Table 2). None of the patients in the Lemaire group experienced complications, while one patient in the Reference group experienced a cyclops syndrome (treated by arthroscopic debridement).

**Table 1:** Demographics and preoperative data

	Lemaire group *n* = 35 knees		Reference group *n* = 52 knees		*p*-value*
	n (%)		n (%)		
	Mean ± SD	Range	Mean ± SD	Range	
**Demographics**					
Male sex	28 (80%)		29 (56%)		0.021
Age *(years)*	23.0 ± 6.3	(15–37)	34.2 ± 10.5	(16–53)	< 0.001
BMI *(kg/m²)*	23.5 ± 2.5	(20–29)	23.9 ± 2.7	(18–31)	n.s.
Tegner score** (pre-injury)	7.5	(7–9)	6.5	(7–9)	0.001
**Preoperative data**					
Lachman					n.s.
Hard endpoint	0 (0%)		0 (0%)		
Delayed hard endpoint	0 (0%)		4 (8%)		
Soft endpoint	35 (100%)		48 (92%)		
Pivot Shift					< 0.001
None	0 (0%)		0 (0%)		
Glide	0 (0%)		51 (98%)		
Clunk	32 (91%)		0 (0%)		
Gross	3 (9%)		0 (0%)		
Lysholm score	65.4 ± 17.9	(35–100)	68.2 ± 16.7	(34–99)	n.s.
IKDC subjective score	61.3 ± 15.0	(23–91)	59.7 ± 13.1	(22–86)	n.s.
Static ATT	2.6 ± 3.7	(5–10)	1.5 ± 2.3	(4–6)	n.s.
Dynamic ATT (Differential laxity)	7.9 ± 3.9	(0–17)	5.9 ± 3.6	(3–14)	0.022
Extension deficit	1 (3%)		1 (2%)		n.s.
Flexion range	135.4 ± 13.7	(60–140)	133.4 ± 7.5	(115–140)	0.022

Abbreviations: *BMI* Body Mass Index, *IKDC* International Knee Documentation Committee, *ATT* Anterior Tibial Translation, *Op* Operated, *Contra* Contralateral

*Fisher’s exact test or Wilcoxon rank sum test; **Tegner score reported as Median and Interquartile range

**Table 2:** Intraoperative data

	Lemaire group *n* = 35 knees		Reference group *n* = 52 knees		*p*-value*
	n	(%)	n	(%)	
**Meniscal lesions**					n.s.
None	16	(46%)	20	(38%)	
Isolated medial	10	(29%)	20	(38%)	
Isolated lateral	3	(9%)	8	(15%)	
Bicompartmental	6	(17%)	4	(8%)	
**Medial meniscal treatment**					n.s.
No lesion	19	(54%)	28	(54%)	
Untreated	0	(0%)	2	(4%)	
Resected	2	(6%)	5	(10%)	
Sutured	14	(40%)	17	(33%)	
**Lateral meniscal treatment**					n.s.
No lesion	26	(74%)	40	(77%)	
Untreated	0	(0%)	2	(4%)	
Resected	4	(11%)	4	(8%)	
Sutured	5	(14%)	6	(12%)	

Abbreviation: *ACL* Anterior Cruciate Ligament

*Fisher’s exact test

### Isokinetic scores

In concentric mode the hamstrings deficit was similar among the two groups at 240°/s (9.0% ± 16.6 vs 10.9% ± 16.6, *p* = 0.910) and at 90°/s (9.0% ± 13.6 vs 8.5% ± 22.4, *p* = 0.993), while the quadriceps deficit was smaller in the Lemaire group than the Reference group at 240°/s (13.6 ± 12.2 vs 18.2 ± 13.4, *p* = 0.127) and at 90°/s (12.2 ± 15.9 vs 15.6 ± 14.1, *p* = 0.141) (Table 3). The only significant difference was the mixed H/Q ratio which was lower in the Lemaire group than in the Reference group (1.02 ± 0.19 vs 1.14 ± 0.24, *p* = 0.011).

**Table 3:** Isokinetic data

	Lemaire group *n* = 35 knees		Reference group *n* = 51 knees		*p*-value*
	Mean ± SD	Range	Mean ± SD	Range	
**Concentric mode 240°/s**					
Hamstrings deficit (%)	9.0 ± 16.6	(−47.0–34.3)	10.9 ± 16.6	(−39.4–66.7)	n.s.
Quadriceps deficit (%)	13.6 ± 12.2	(− 21.9–45.6)	18.2 ± 13.4	(−9.3–43.1)	n.s.
**Concentric mode 90°/s**					
Hamstrings deficit (%)	9.0 ± 13.6	(− 16.6–31.6)	8.5 ± 22.4	(− 63.4–85.3)	n.s.
Quadriceps deficit (%)	12.2 ± 15.9	(− 21.7–58.3)	15.6 ± 14.1	(−15.9–46.7)	n.s.
**Eccentric mode 30°/s**					
Hamstrings deficit (%)	12.0 ± 17.1	(−42.7–43.0)	11.2 ± 13.5	(−15.0–45.4)	n.s.
**H/Q Ratios**					
Concentric 240°	0.64 ± 0.15	(0.41–1.14)	0.66 ± 0.15	(0.19–1.16)	n.s.
Concentric 90°	0.60 ± 0.12	(0.36–1.02)	0.62 ± 0.15	(0.08–0.90)	n.s.
Mixed†	1.02 ± 0.19	(0.61–1.50)	1.14 ± 0.24	(0.71–1.77)	0.011

Abbreviations: *H* Hamstrings, *Q* Quadriceps

*Wilcoxon rank sum test

†Eccentric Hamstrings at 30°/s ÷ Concentric Quadriceps at 240°/s

### Clinical outcomes

Lysholm, IKDC and ACL-RSI were similar among the two groups (Table 4). Tegner scores were however higher for the Lemaire group than the Reference group (median, 6.5 vs 6.0, *p* = 0.024), but the net decrease in Tegner score was equivalent among the two groups (median, 1.0 vs 1.0). The two groups had a similar flexion range (*p* = 0.174), with no extension deficits in either group (*p* = 0.915). A glide pivot-shift was observed in one patient (3%) in the Lemaire group, but in none of the patients in the Reference group.

**Table 4:** Postoperative data

	Lemaire group *n* = 35 knees		Reference group *n* = 51 knees		*p*-value*
	n (%)		n (%)		
	Mean ± SD	Range	Mean ± SD	Range	
**Postoperative data**					
Lachman					0.025
Hard endpoint	33 (94%)		51 (100%)		
Delayed hard endpoint	2 (6%)		0 (0%)		
Soft endpoint	0 (0%)		0 (0%)		
Pivot Shift					n.s.
None	34 (97%)		51 (100%)		
Glide	1 (3%)		0 (0%)		
Clunk	0 (0%)		0 (0%)		
Gross	0 (0%)		0 (0%)		
Complications	0 (0%)		1 (2%)		n.s.
Tegner score**	6.5	(6–8)	6.0	(5–7)	0.024
Lysholm	90.2 ± 12.1	(34–100)	90.1 ± 6.2	(75–100)	n.s.
IKDC subjective score	88.4 ± 11.6	(39–100)	85.9 ± 8.8	(59–98)	n.s.
ACL-RSI	77.1 ± 14.5	(33–96)	78.7 ± 11.5	(42–98)	n.s.
Extension deficit	0 (0%)		2 (4%)		n.s.
Flexion range	137.6 ± 3.7	(130–140)	136.1 ± 5.7	(115–140)	n.s.
**Net change**					
Tegner score**	− 1.0	(−2–0)	−1.0	(−2–0)	n.s.
Lysholm	24.8 ± 20.0	(30–60)	22.3 ± 17.5	(14–57)	n.s.
IKDC subjective score	27.1 ± 18.7	(24–72)	26.4 ± 13.1	(1–53)	n.s.

Abbreviations: *IKDC* International Knee Documentation Committee, *ACL-RSI* Anterior Cruciate Ligament Return to Sport after Injury

*Fisher’s exact test or Wilcoxon rank sum test; **Tegner score reported as Median and Interquartile range

## Discussion

The most important finding of this study was that ACLR with a modified Lemaire procedure for knees with rotational instability could provide similar isokinetic muscle recovery as does stand-alone ACLR in knees with minimal rotational instability. The hypothesis is therefore confirmed, suggesting that, for ACL-deficient knees with high-grade pivot-shift, a modified Lemaire procedure restores rotational stability without increasing isokinetic muscle deficit at the time of RTP.

In the present series, none of the patients had high-grade pivot-shift at 8 months following ACLR, and only one knee (3%) in the Lemaire group had low-grade residual pivot-shift. This corroborates the findings of systematic reviews that found LET procedures to reduce pivot-shift, leading to greater rotational stability [21, 38]. Furthermore, this series had no graft failures in the Lemaire group, compared to 1 graft failure in the Reference group. This is in agreement with studies which found that a LET does not increase risks of graft failure [37, 42], and could in fact protect the graft in the early postoperative period by decreasing the forces on it by 43% and by addressing the residual instability as a risk for graft failure [1, 18].

The isokinetic strength deficits observed in the Lemaire group of this study are comparable to isokinetic strength deficits reported for stand-alone ACLR (without LET) in the literature. Tashiro et al. [40] found, using lower angular velocities, a quadriceps deficit of 20% at 60°/s, and 10% at 180°/s. Yosmaoglu et al. [45] found quadriceps deficit of 18.3% at 60°/s, and 15.3% at 180°/s, and a hamstrings deficit 21.3% at 60°/s, and 9.6% at 180°/s. Kyung et al. [24] found, using semitendinosus and gracilis graft, quadriceps deficits of 29.9% at 60°/s and 18.5% at 180°/s, and hamstrings deficits of 24.6% at 60°/s and 10.5% at 180°/s.

Isokinetic evaluation of athletic patients is an integral part in the rehabilitation program and to validate RTP [31, 43, 44]. The findings of this study revealed similar isokinetic test outcomes at all speeds and modes, but a significant difference in the mixed H/Q ratio (*p* = 0.011) (Table 3). Croisier et al. [10] advocate that mixed H/Q ratios are the most important since they closely represent real biomechanical conditions of athletes during contact sports, particularly actions such as sprinting and kicking.

The indications and techniques for extra-articular procedures during ACLR are constantly evolving [16]. Recently, several authors revised the indications for extra-articular procedures to patients younger than 30 years or those with concomitant medial meniscus tears [6, 35]. The biomechanical function and benefits of LET have been widely studied, demonstrating its role of limiting translation within the lateral compartment [15, 27, 39, 47], reducing pivot-shift [16, 30, 32, 47], and protecting the graft and meniscus [17, 18, 30], with little or no side effects [36]. Some authors found that LET improves clinical outcomes [27, 34, 46], while in this study, the postoperative functional outcomes are equivalent between the Lemaire group and Reference group, despite greater preoperative laxity (*p* = 0.022) and rotational instability (*p* < 0.001) in the Lemaire group (Table 1).

The authors believe that the terminology used to describe extra-articular procedures should be reconsidered. This is because the general term ‘lateral extra-articular tenodesis’ could be misleading, as the procedure does not involve suturing the end of a tendon to a bone. The term ‘lateral extra-articular plasty’ better describes the moulding of the tensor fascia latae, which is not a tendon, and therefore exhibits more elastic properties.

This study has several limitations which must be taken into account. The number of patients in each cohort was small, and due to the specific indications for a Lemaire procedure, the demographics were not equivalent; the Lemaire group was 10 years younger, had a greater proportion of males and higher Tegner scores. Younger patients are typically more active and potentially more motivated to return to activity. As Tegner score was significantly different, it was opted to compare the isokinetic strength deficits instead of the absolute strength. A control group of patients with severe rotational knee instability was not included because of the indications for a Lemaire procedure. Furthermore, the groups also differed in meniscal lesions and treatments. In addition, isokinetic deficits were tested at 240°/s and 90°/s, validated by Burgi et al. [4] and Undheim et al. [43], while most studies used 180°/s and 60°/s. Nevertheless, this study has several strengths, including the consistency of surgical technique and graft type, as well as being the first to investigate the effect of a Lemaire procedure on isokinetic muscle recovery.

## Conclusion

ACLR with a modified Lemaire procedure for knees with rotational instability grants equivalent isokinetic muscle recovery as does stand-alone ACLR in knees with no rotational instability. For ACL-deficient knees with high-grade pivot-shift, a modified Lemaire procedure restores rotational stability without compromising isokinetic muscle recovery at the time of return-to-play.

## Supplementary Information

**Additional file 1.**

## Data Availability

Data are available upon reasonable request.
