# Supplementary material for: Adding a modified Lemaire procedure to ACLR in knees with severe rotational knee instability does not compromise isokinetic muscle recovery at the time of return-to-play
Source: J Exp Orthop. 2020 Oct 30;7:84. doi: 10.1186/s40634-020-00302-1 (PMC7599282; doi:10.1186/s40634-020-00302-1)
Supplement: Supplementary file 1 — Additional file 1. [file 40634_2020_302_MOESM1_ESM.docx]

*Postoperative rehabilitation*

The rehabilitation protocol was divided into three phases:

Phase 1 (0-6 weeks): The aims were to eliminate effusion, pain, range of motion (ROM) deficits and minimize muscle atrophy. The ROM goals were to obtain full extension and 90° of flexion at 3 weeks, then 120° of flexion at 6 weeks. The rehabilitation program consisted of daily quadriceps contractions, ROM exercises, patella mobilization, concentric and eccentric exercises at low velocity and stationary cycling as soon as tolerated. Partial weight-bearing (50% body weight) was allowed during the first 3 postoperative weeks if the preoperative static ATT was < 5mm,[13] and progressive full weight-bearing was allowed between 3 and 6 weeks.

Phase 2 (6-12 weeks): The aims were to get full ROM, increase muscular strength and knee stability. Rehabilitation program insisted of quadriceps and hamstrings stretching exercises, with progressive introduction of neuromuscular training and strength training. At the end of this phase, the patient was expected to walk quickly and climb stairs.

Phase 3 (3-8 months): The aim was to regain the normal muscle strength and gradual return to sport. This phase consisted of heavy resistance strength training and exercises depending on type of sport practiced. Plyometric exercises were introduced with increasing intensity.
